# Patient‐reported pain severity and health‐related quality of life in patients with multiple myeloma in real world clinical practice

**DOI:** 10.1002/cnr2.1429

**Published:** 2021-06-10

**Authors:** Heinz Ludwig, Abigail Lucy Bailey, Andrea Marongiu, Keerun Khela, Gary Milligan, Katherine Brewer Carlson, Alex Rider, Anouchka Seesaghur

**Affiliations:** ^1^ Medical Department Center for Oncology, Hematology and Palliative Medicine Wilhelminen Cancer Research Institute Vienna Austria; ^2^ Oncology Adelphi Real World, Adelphi Mill Bollington UK; ^3^ Centre for Observational Research Amgen Ltd Uxbridge UK; ^4^ Center for Observational Research Amgen Inc Thousand Oaks California USA

**Keywords:** pain, patient‐reported, quality of life

## Abstract

**Background:**

The association between patient self‐reported pain severity and health‐related quality‐of‐life (HRQoL) is poorly understood.

**Aims:**

This real‐world study of symptomatic multiple myeloma (MM) patients sought to determine how pain severity from a single question asked during routine clinical consultation was associated with HRQoL.

**Methods and results:**

Point‐in‐time data on HRQoL of 330 patients with MM (median age 70 years) receiving anti‐myeloma therapy in Germany and Italy from November 2017 through February 2018 were analyzed. HRQoL was assessed using validated questionnaires (Work Productivity and Activity Impairment [WPAI], European Organization for Research and Treatment of Cancer Quality of Life Questionnaire ‐C30 and ‐MY20). Physical pain severity was assessed during clinical consultation by a single question, asking patients to describe their pain as “no pain,” “mild,” “moderate,” or “severe.” Associations between patient‐reported pain severity and HRQoL scores were assessed by analysis of variance or *χ* ^2^ tests. Ninety‐six of the 330 patients (29.1%) reported moderate to severe pain. Increase in pain severity, from “no” to “severe” pain, was associated with significantly decreased overall HRQoL (mean score 70.2 to 33.3); significant decreases in levels of physical (82.7 to 35.1), social (81.1 to 44.4), emotional (78.1 to 48.3), and role functioning (79.5 to 38.9); and increased levels of WPAI usual activity impairment (35.4 to 71.4), and fatigue burden (26.0 to 68.9) (all *p* < .001).

**Conclusion:**

Higher pain severity, based on a single self‐report question, was associated with poorer HRQoL in patients with MM, thereby supporting the clinical relevance of directly asking patients to self‐evaluate their pain severity.

## 1 INTRODUCTION

Pain is a common debilitating symptom of cancer and contributes to poor health‐related quality of life (HRQoL).^1^ Patients with multiple myeloma (MM), especially those with relapsed/refractory disease, experience significantly more pain and poorer HRQoL (including physical, role, emotional, and social functioning) compared to an age‐matched general population of the same geographical region.^2^

Pain in MM is often due to myeloma bone disease, which is observed in more than 80% of patients during the course of their disease. Patients with earlier manifest or new bone disease experience discomfort or pain and a decrease in HRQoL. With effective myeloma therapy, bone pain and other myeloma‐associated pain usually improves or resolves,^3^ especially in patients with deep response to induction therapy and during maintenance phase.^2^ Most patients unfortunately relapse after a variable length of time following successful induction therapy. A recent systematic review and meta‐analysis suggest that pain is particularly prominent during uncontrolled disease at start of initial therapy and at relapse.^4^ These patients require both optimal MM therapy and efficient pain management.^3^

Although pain management is an integral part of cancer care, pain is underreported, misunderstood, and often undertreated.^5^ A systematic review of 20 articles published from 2007 to 2013 revealed that 31.8% of patients with cancer were not receiving pain medication proportional to their pain intensity.^6^ This suggests both suboptimal attention to the patient's symptoms and difficulties in assessing pain in routine practice. To provide care that is meaningful to patients with MM, capturing and understanding the patient perspective are therefore essential. Thus, every effort should be taken to integrate patient‐reported HRQoL into real‐life MM treatment and this principle should not be hindered by cost, training, physician ability to recognize symptoms, and logistical considerations.^7^, ^8^

Information on HRQoL is important to understand the patient's symptom burden and needs. While several valid and reliable tools exist for assessment of pain severity, these tools are established in clinical trials and outcome research but not commonly used in routine clinical care, where they may be impractical due to time constraints and other reasons.^9^ A simple approach to quantify pain severity in patients with MM is via self‐reported assessment during routine consultation.

In this study, we examined whether patients' self‐reported pain severity based on a single question relates to the magnitude of changes in QoL items such as HRQoL, functional and emotional impairment, fatigue, and work productivity in a real‐world clinical setting. To our knowledge, no published study has examined the association between pain severity in patients with MM and specific other domains of HRQoL.

## 2 METHODS

A point‐in‐time study was conducted using the Adelphi Multiple Myeloma Disease Specific Programme (DSP) patient‐level database. Details of the full DSP methodology have been described previously.^10^ This study was an analysis of secondary data collected between November 20, 2017 and February 1, 2018 from hospital and office settings in Germany and Italy.

### 2.1 Participants and measures

Consenting adults (≥18 years at time of survey) with symptomatic MM and who received either first‐line or second/subsequent‐line MM therapy voluntarily completed questionnaires independent of their treating physician at routine face‐to‐face consultations (referred to as time of survey) and were returned in a sealed envelope to ensure confidentiality. Each physician invited their next eight consecutively consulting patients to participate in the study to minimize selection bias.

Patients were asked to rate their pain severity using a single question: “Please tick the box that best describes the level of pain that you are currently experiencing.” Each patient chose one response: “no pain,” “mild pain,” “moderate pain,” or “severe pain.” Responses to this question were aligned with responses from the validated EuroQol 5‐dimension 5‐level (EQ‐5D‐5L) questionnaire on pain/discomfort^11^; responses to the single question were also concordant with the relevant responses from the EQ‐5D‐5L (Appendix S1). In addition, responses to the European Organization for Research and Treatment of Cancer Quality of Life (EORTC QLQ) 20‐item myeloma‐specific questionnaire (EORTC QLQ‐MY20)^12^ on bone aches/pain (question 31) were also aligned with the single pain question (Appendix S1).

The EORTC QLQ‐MY20 asked patients “During the past week, have you had the following symptoms or problems.” Patients were considered to have bone pain in the past 7 days before the survey if they answered “A Little,” “Quite a Bit” or “Very Much” to any combination of EORTC QLQ‐MY20 questions 31 to 33, and 35 on bone pain/aches (question 31) or site‐specific bone pain (questions 32‐33, 35). Patients were considered to have bone pain/aches (non‐site specific) based on positive responses to question 31, and site‐specific bone pain (back, hip, and chest) based on responses to questions 32, 33, and 35, respectively.

Several dimensions of HRQoL (physical, role, emotional, and social functioning and fatigue) were assessed via the validated EORTC Core‐30 Questionnaire version 3 (EORTC QLQ‐C30).^13^ Work productivity and activity impairment were assessed using the validated Work Productivity and Impairment questionnaire (WPAI).^14^ Table S1 presents a short description of the validated questionnaires used.

Detailed information on patient demographics, diagnosis, clinical status, concomitant conditions, current treatments (including analgesics) at the time of survey, and treatment history were available from medical records reported by treating physicians (hematologists or hematology‐oncologists) via electronic standardized forms. The stage of disease at the time of survey was classified using the Multiple Myeloma International Staging System (ISS).^15^

### 2.2 Ethics

Physicians consented to participate and provide patient medical information during screening. Patients provided informed consent prior completion of questionnaires, with all data aggregated and de‐identified before receipt. DSP data were collected according to procedures established at Adelphi Real World and are compliant with the European Pharmaceutical Market Research Association Code of Conduct, the Health Information Technology for Economic and Clinical Health Act^16^ and the Health Insurance Portability and Accountability Act^17^ as appropriate in each specific region or territory. International approval for the survey was also granted by the Freiburger Ethik Kommission International.

### 2.3 Statistical analysis

Descriptive statistics are reported for demographics, clinical characteristics, and patient‐reported outcomes (PRO). Frequency and percentages are reported for categorical variables and mean and SD or median and interquartile range (IQR) for continuous variables. Comparisons between pain severity categories (no pain, mild pain, moderate pain, and severe pain) were performed by *χ* ^2^ test for categorical variables and analysis of variance (ANOVA) for continuous variables. *p*‐values <.05 were considered statistically significant. Additionally, for some continuous outcomes, univariate linear regressions were conducted to show pairwise comparisons between no pain and each of the other three pain groups (mild, moderate, severe).

Data were imputed for some PRO domains as instructed by the tool authors. Beyond that, incomplete or missing data were not imputed. Each individual estimate (such as mean or percent) were calculated based on available data and therefore patient numbers varied across analyses. The number of missing patients were included with patient demographic or clinical results as appropriate.

Statistical analyses were performed using STATA statistical software version 15.1 (StataCorp LLC, College Station, Texas).

## 3 RESULTS

### 3.1 Patient demographic and clinical characteristics

Three‐hundred‐thirty MM patients were included; most (*n* = 267; 80.9%) were from Germany. Approximately two‐thirds were female (*n* = 207; 62.7%) and similar proportions were retired at the time of survey (*n* = 216; 65.5%). Median age was 70 years (IQR: 62‐75 years) (Table 1).

**TABLE 1:** Demographic and clinical characteristics of multiple myeloma patients stratified by self‐reported pain severity

Demographic and clinical characteristics	All patients	Patient self‐reported pain severity at time of survey			
		No pain	Mild pain	Moderate pain	Severe pain
Total, *n* (%)	330 (100)	73 (22.0)	161 (48.8)	81 (24.6)	15 (4.6)
Country					
Germany	267 (80.9)	58 (79.5)	131 (81.4)	64 (79.0)	14 (93.3)
Italy	63 (19.1)	15 (20.5)	30 (18.6)	17 (21.0)	1 (6.7)
Age, years—median (Q1, Q3)	70 (62, 75)	64 (57, 72)	70 (63, 75)	72 (66, 76)	71 (67, 77)
Age group					
18–44 years	8 (2.4)	3 (4.1)	3 (1.9)	2 (2.5)	0 (0.0)
45–64 years	96 (29.1)	34 (46.6)	45 (28.0)	16 (19.8)	1 (6.7)
≥65 years	226 (68.5)	36 (49.3)	113 (70.2)	63 (77.8)	14 (93.3)
Female, *n* (%)	207 (62.7)	46 (63.0)	104 (64.6)	47 (58.0)	10 (66.7)
Working, *n* (%)					
Full/part‐time	65 (19.6)	23 (31.5)	34 (21.1)	7 (8.7)	1 (6.7)
Retired	216 (65.5)	37 (50.7)	107 (66.5)	59 (72.8)	13 (86.7)
Time since diagnosis, years—median (Q1, Q3)	0.96 (0.30, 2.07)	1.26 (0.38, 3.02)	1.08 (0.30, 2.07)	0.66 (0.26, 1.79)	0.41 (0.28, 1.0)
International Staging System stage, *n* (%)					
Stage I	65 (19.7)	20 (27.4)	33 (20.5)	9 (11.1)	3 (20)
Stage II	88 (26.7)	15 (20.5)	40 (24.8)	26 (32.1)	7 (46.7)
Stage III	160 (48.5)	29 (39.7)	85 (52.8)	42 (51.9)	4 (26.7)
Unknown/not assessed	17 (5.2)	9 (12.3)	3 (1.9)	4 (4.9)	1 (6.7)
Line of anti‐myeloma therapy, *n* (%)					
First line	175 (53.0)	41 (56.2)	79 (49.1)	45 (55.6)	10 (66.7)
Second line	141 (42.7)	28 (38.4)	74 (46.0)	34 (42.0)	5 (33.3)
Third line	6 (1.8)	2 (2.7)	3 (1.9)	1 (1.2)	0 (0)
Fourth line or later	8 (2.4)	2 (2.7)	5 (3.1)	1 (1.2)	0 (0)
Eastern Cooperative Oncology Group performance status, *n* (%)					
0—fully active	91 (27.6)	39 (53.4)	39 (24.2)	12 (14.8)	1 (6.7)
1—restricted	157 (47.6)	30 (41.1)	87 (54.0)	31 (38.3)	9 (60)
2—unable to work	65 (19.7)	4 (5.5)	27 (16.8)	30 (37.0)	4 (26.7)
3 or 4—limited self‐care/bed‐bound	17 (5.2)	0 (0)	8 (5.0)	8 (9.9)	1 (6.7)
Main comorbidities at time of survey^a^, *n* (%)					
None	91 (27.6)	34 (46.6)	40 (24.8)	14 (17.3)	3 (20.0)
Cardiovascular	169 (51.2)	23 (31.5)	87 (54.0)	59 (61.5)	48 (59.3)
Hypertension	122 (37.0)	15 (20.5)	67 (41.6)	40 (41.7)	35 (43.2)
Metabolic	96 (29.1)	11 (15.1)	57 (35.4)	28 (29.2)	26 (32.1)
Organ disease	66 (20)	11 (15.1)	36 (22.4)	5 (5.2)	18 (22.2)
Diabetes	58 (17.6)	7 (9.6)	35 (21.7)	15 (18.5)	1 (6.7)

^a^ Patients may have more than one comorbidity each.

The median time (IQR) between MM diagnosis and the time of survey was 0.96 (0.30, 2.07) years. Nearly half (48.5%) of patients with MM had lived longer than 1 year with MM and 17.3% had lived with MM for more than 3 years (data not shown). The most common comorbidity was cardiovascular disease (*n* = 169; 51.2%) (Table 1). Almost half of patients had ISS stage III disease (*n* = 160; 48.5%), and about half (46.9%) of patients were receiving second‐line treatment or higher. A quarter of patients (*n* = 82; 24.8%) had moderate‐to‐severe performance status (ie, unable to work or bedridden [Eastern Cooperative Oncology Group—ECOG performance status ≥2]; Table 1). Overall, combination chemotherapy was the most common treatment received at time of survey across all lines of therapy, with 145 (43.9%) and 141 (42.7%) patients on lenalidomide‐ and bortezomib‐based regimens, respectively.

### 3.2 Bone health

Most patients (*n* = 289; 87.6%) reported bone pain (non‐site specific and site‐specific) at the time of survey. Over three‐quarters (*n* = 252; 76.4%) of patients had self‐reported non‐site‐specific bone aches or pain. Vertebral (back) pain was reported by most patients (*n* = 238; 72.1%). Other sites of bone pain were hip (*n* = 185; 56.1%) and chest/rib (*n* = 110; 33.3%) (Table 2).

**TABLE 2:** Bone health and management of multiple myeloma patients stratified by patient self‐reported pain severity

	Patient self‐rated pain severity at time of survey				
	All patients	No pain	Mild pain	Moderate pain	Severe pain
Total, *n* (%)	330	73	161	81	15
Bone pain (self‐reported) 7 days prior to survey^a^, *n* (%)					
No bone pain	41 (12.4)	33 (45.2)	7 (4.3)	1 (1.2)	0 (0.0)
Bone pain (overall)^b^	289 (87.6)	40 (54.8)	154 (95.7)	80 (98.8)	15 (100.0)
Bone pain or aches (non‐site specific)	252 (76.4)	26 (35.6)	132 (82.0)	79 (97.5)	15 (100.0)
Bone pain—site specific^c^					
Vertebral (back) pain	238 (72.1)	20 (27.4)	126 (78.3)	78 (96.3)	14 (93.3)
Non‐vertebral pain	215 (65.2)	27 (37.0)	106 (65.8)	68 (84.0)	14 (93.3)
Chest/rib pain	110 (33.3)	18 (24.7)	53 (32.9)	28 (34.6)	11 (73.3)
Hip pain	185 (56.1)	18 (24.7)	90 (55.9)	66 (81.5)	11 (73.3)
Record of bone targeting agent use, *n* (%)^d^, ^e^					
Prior to survey	182 (55.2)	28 (38.4)	97 (60.2)	51 (63.0)	6 (40.0)
At time of survey	181 (54.8)	28 (38.4)	97 (60.2)	50 (61.7)	6 (40.0)
Analgesics at time of survey, *n* (%)					
Missing value	191 (57.9)	62 (84.9)	91 (56.5)	31 (38.3)	7 (46.7)
No analgesic use	8 (2.4)	1 (1.4)	7 (4.3)	0 (0.0)	0 (0.0)
Analgesic use	131 (39.7)	10 (13.7)	63 (39.1)	50 (61.7)	8 (53.3)
Non‐opioid analgesics	69 (20.9)	10 (13.7)	40 (24.8)	16 (19.8)	3 (20.0)
Weak opioids	36 (10.9)	0 (0.0)	13 (8.0)	21 (25.9)	2 (13.3)
Strong opioids ≤75 mg OME per day	23 (7.0)	0 (0.0)	8 (5.0)	12 (14.8)	3 (20.0)
Strong opioids >75–150 mg OME per day	3 (0.9)	0 (0.0)	2 (1.2)	1 (1.2)	0 (0%)

*Note*: All values *n* (%) unless otherwise indicated.

Abbreviations: MM, multiple myeloma; OME, oral morphine equivalent; SRE, skeletal‐related event.

^a^ Patient‐reported data.

^b^ Response to one or more EORTC QLQ‐MY20 questions 31, 32, 33, or 35 on bone aches/pain or site‐specific pain.

^c^ Patients may have reported bone pain in one or more sites.

^d^ Patients may have received more than one different type of bone health agent.

^e^ Bone health agents include denosumab, zoledronic acid, pamidronic acid, clodronic acid, ibandronic acid.

Over half of the patients (*n* = 182; 55.2%) had a record of bone‐targeting agents (BTA). Of the 289 (87.6%) patients with self‐reported bone pain, 166 (57.4%) had received a BTA (data not shown); of the 73 (22.1%) patients not experiencing pain, 28 (38.4%) had received a BTA.

At the time of survey, 39.7% were receiving analgesics, 52.7% of whom were on non‐opioid, 27.5% on weak opioids, and 19.8% on strong opioids (Table 2).

### 3.3 Patient‐reported pain severity

Patients reported their current level of pain severity at the time of survey via the single question as no pain, mild, moderate, or severe pain by 73 (22.0%), 161 (48.8%), 81 (24.6%), and 15 (4.6%) patients, respectively (Table 1). Demographic and clinical characteristics of patients when stratified by self‐reported pain severity are shown in Table 1. Both countries had similar proportions of patients in each pain group (data not shown).

Among those reporting “no pain” at the time of survey (*n* = 73), about half were <65 years (50.7%), had an ECOG status of 0 (53.4%), and had no record of active comorbidities (46.6%) (Table 1). Around 55% of these patients reported experiencing bone pain during the past 7 days prior to the survey (Table 2); of those with bone pain, 28 (70%) patients had a history of BTA use and 9 (23%) had a history of analgesics use at time of survey (data not shown). Use of analgesics increased from 13.7% in patients without self‐reported pain to 53.3% in those who self‐reported severe pain (Table 2).

Most of the patients (>95%) who reported experiencing pain (of any intensity; mild, moderate or severe pain) at the time of survey had suffered bone pain (either bone aches or pain or site‐specific bone pain) in the 7 days before the survey, with back pain being the most common (Table 2). All patients who self‐reported severe pain (100%) had informed their physicians that they experienced bone pain in the past 7 days before the survey. Similarly, nearly all patients who self‐reported moderate (*n* = 80/81; 98.9%) or mild pain (*n* = 154/161; 95.7%) also reported bone pain (Table 2). Among the 73 patients who self‐reported “no pain,” nearly half (45.2%) did not report experiencing bone pain in the past 7 days before the survey. The remaining patients (*n* = 40/73; 54.8%) recalled experiencing bone pain, with some specifying the site (Table 2).

### 3.4 Patient‐reported pain severity and HRQoL

**FIGURE 1:**
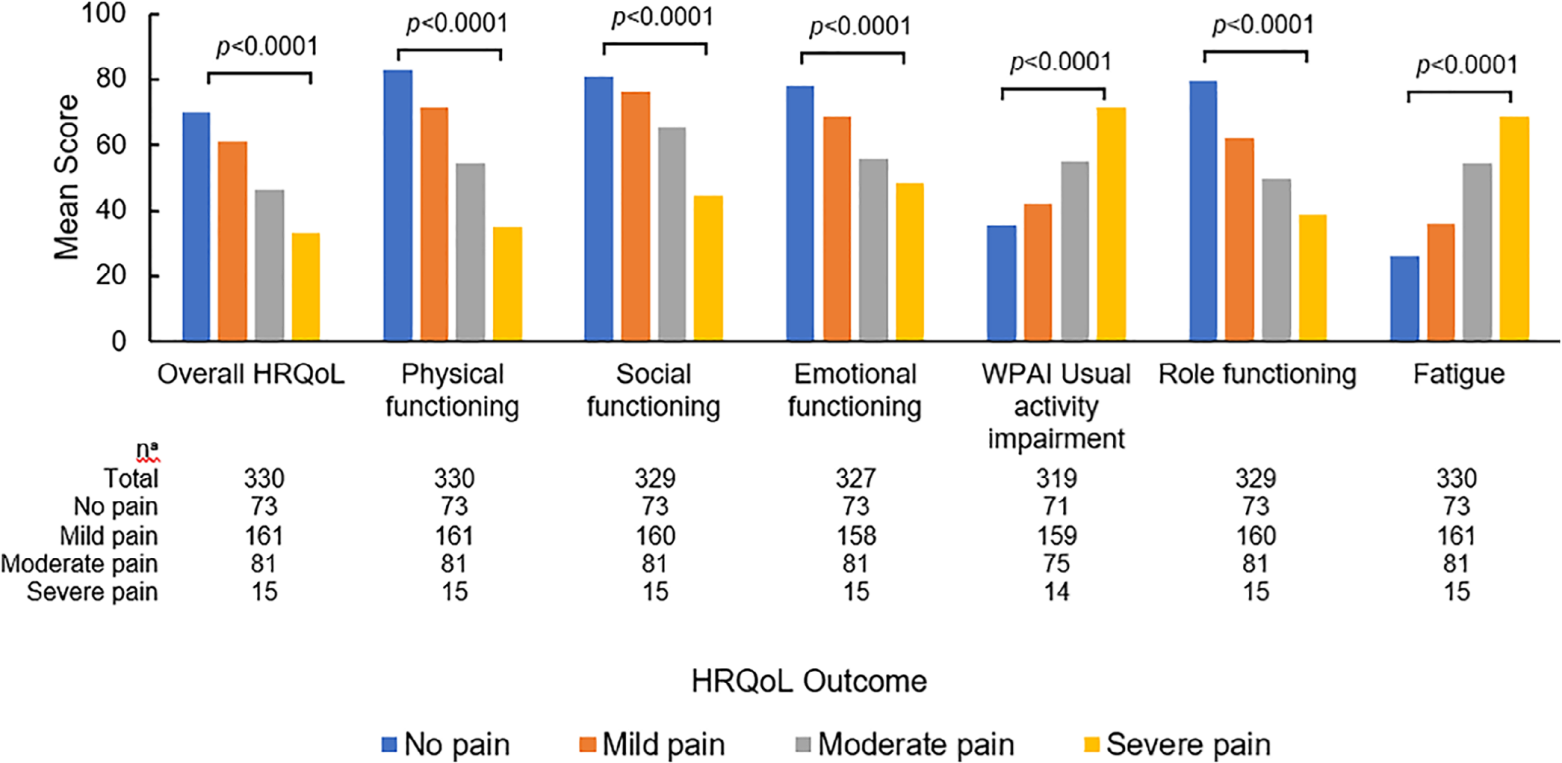
Associations between patient self‐reported pain severity and health‐related quality‐of‐life outcomes. *p*‐values are based on ANOVA. *Refer to Table S1 for relevant information on validated PRO questionnaires. EORTC QLQ‐C30 and WPAI usual activity impairment scores shown. ^a^Bases for each PRO score relating to patient pain severity at time of survey

To assess the association between pain severity and different HRQoL outcome measures, patients were stratified according to their current level of self‐reported pain severity at the time of survey (defined as “no pain”, “mild pain”, “moderate pain” or “severe pain”). As the level of patient self‐reported pain severity increased from no pain to severe pain, mean scores on overall HRQoL decreased from 70.2 to 33.3; this association was statistically significant (ANOVA; *p* < .001) (Figure 1). A higher level of self‐reported pain severity was associated with poorer physical, social, and emotional functioning. As pain severity increased from no pain to severe pain, we observed decreases in EORTC QLQ‐C30 mean scores on physical (82.7 to 35.11), social (81.1 to 44.4), emotional (78.1 to 48.3), and role (79.5 to 38.9) functioning (ANOVA; all *p* < .001). Means scores for WPAI usual activity impairment (35.4 to 71.4) and EORTC QLQ‐C30 fatigue burden (26.0 to 68.9) increased (ANOVA; all *p* < .001) as the level of patient self‐reported pain severity rose (Figure 1).

Overall work productivity loss was based on a subset of 42 patients in full‐ or part‐time employment who completed the WPAI questionnaire. As the level of patient self‐reported pain severity increased, we observed increases in both the mean percentage of work time missed (absenteeism; *p* = .06) and the mean percentage of impairment experienced while at work (presenteeism; *p* = .04) (data not shown).

### 3.5 Increasing pain severity associated with reductions in HRQoL and work productivity

Table S2 shows the linear regression coefficients for the association between the level of patient self‐reported pain severity and HRQoL. In all patients, we observed a significant association between patients self‐reported pain and HRQoL. Mean HRQoL differences between no pain and the other three pain groups were significant (*p* < .05) in all instances, with the exception of activity impairment due to a problem and social functioning in those with self‐reported mild pain. These results indicate that increasing self‐reported pain was associated with poorer overall health status and functioning, increased work and activity impairment, and fatigue.

## 4 DISCUSSION

This real‐world study examined how patients' self‐reported pain severity, from a single question asked during clinical consultation, is associated with HRQoL in symptomatic MM patients receiving anti‐myeloma therapy in routine practice. Higher pain severity self‐reported by patients with MM via the single question was associated with significantly poorer overall HRQoL, poorer physical, social, and emotional functioning, and greater functional impairment and fatigue burden.

Although MM remains a largely incurable condition, the introduction of novel therapies has significantly improved clinical outcomes. For many patients, MM is a long‐term chronic condition characterized by a remitting and relapsing course.^18^ A study on MM patients in England across all disease stages revealed that general symptom level, pain, and anxiety predicted declining HRQoL.^19^ Previous research has shown that higher grades of chemotherapy induced peripheral neuropathy were correlated with worse HRQoL outcomes,^20^ and that patients with MM experience a high symptom burden and low HRQoL.^21^ Therefore, an accurate and thorough assessment of pain in MM is crucial for identifying the underlying etiology and for developing a treatment plan.

An accepted standard of care, recently reinforced by a guide from the European Society for Medical Oncology, is that healthcare professionals should routinely assess patient's cancer pain by asking them to rate their pain on a scale of 0 to 10, where 0 is no pain at all and 10 is the worst pain imaginable. Patients who find it difficult to give their pain intensity a number are asked to use linear analogue scales, which allows them to mark their individual pain severity between the extremes of no pain and very severe pain.^22^ In our study, responses to a single question were consistent with responses to validated questionnaires (EQ‐5D‐5L, EORTC QLQ‐MY20), indicating that a straightforward single question can provide an accurate perspective of the patient's pain severity.

Due to the lack of common sampling across studies and region, interpretation of the patient‐reported outcomes data from our study compared to other published studies is limited. A recent study^23^ systematically collected EORTC QLQ‐C30 data from 11 European countries, Canada, and USA to obtain a reference norm for the general population. When compared with the reference European population in that study, our overall sample scored between 10% and 35% poorer (lower for global health, and physical, social, emotional, and role functioning; higher for fatigue). This difference is primarily explained by the scores reported by patients in the moderate and severe pain groups. In addition, our sample is on average older than the reference population.^23^

This point‐in‐time study was based on a sample of MM patients with different disease stages, with or without comorbidities who were treated in routine clinical settings. This lends greater representativeness to real‐world settings than findings based on samples from interventional studies. However, due to the heterogeneous nature of the population in this study, characteristics such as age, gender, specific comorbidities (such as cardiovascular or metabolic), and receipt of analgesics or BTA may act as confounders. Such factors were not assessed within this study due to data on bone complications and pain management not being available for all patients, which precluded the undertaking of multivariate analysis as this would not generate meaningful insights. Many of the analyses conducted in this study produced strongly significant results (*p* < .001), suggesting that the characteristics would need to make a big difference to impact results. Despite these limitations, we observed statistically significant associations between patient‐reported pain severity and HRQoL. In addition, observations from a previous study is consistent with our findings. Ramsentaler et al. reported that clinically relevant anxiety and presence of pain were found to be more predictive of poor or declining HRQoL than demographic and disease clinical parameters, and that treatment history and age were not significantly associated with the HRQoL trajectory of MM patient.^19^ An extensive literature review did not reveal consistent findings regarding age differences in pain sensitivity, but females seem to be more sensitive to both clinical and experimental pain.^24^

Data on bone complications were not available for all patients included in this study. This observation is consistent with previous studies reporting that bone complications are often underestimated from a physician perspective.^25^ Use of BTA was also not available for all patients. Previous research has shown that their impact on severity of bone pain is rather limited.^26^ Moreover, data on pain management (analgesics or BTAs) was not available for all patients. Therefore, it was not possible to understand the true impact of underlying myeloma bone disease on the patient's pain severity^22^, ^27^ or to consider the impact of these medications on the pain severity experienced by the patients.

As this was an analysis of secondary data, the base size of certain subgroups was small, such as those with “severe pain” and those in full‐ or part‐time employment. Data derived from small sample sizes should be interpreted with caution. Larger samples are needed to confirm our results in these subgroups with smaller bases. Approximately 40% of patients invited to participate in the survey by their consulting physicians took part in the survey. Because the survey respondents may differ from the wider MM population, results from this study may not be generalizable to all patients with MM. We saw marginally more female participants in this survey (*n* = 207, 62.7%) than males. The median age of our patient population was 70 years, an age where the differences in the prevalence and life expectancy between males and females becomes relevant. Patients were voluntarily participating in the study, some surveys have shown a greater readiness of females to participate.^28^ In addition, our study is based on data collected at a specific point in time, namely at single face‐to‐face consultation. Although this reduces recall bias of patients' responses to the questionnaires, it does not provide insights on changes in pain severity and HRQoL over the disease course. This calls for further research to investigate changes in the patient's pain experience and HRQoL as disease progresses through intervals of active (often prone to side effects) treatment and stable, treatment‐free phases.

This study demonstrates that the use of a single question can capture the patient's perspective on pain and HRQoL. We observed that a higher level of patient self‐reported pain severity was related to poorer HRQoL. The relationships identified here between patient self‐reported pain severity and HRQoL support the clinical relevance of directly asking patients to self‐rate their pain severity. Hence, a simple and direct approach for understanding pain severity may simplify and expedite HRQoL assessment and may also guide physicians in choosing treatment options to reduce and alleviate pain and improve HRQoL in patients with MM.

## CONFLICT OF INTEREST

H.L. is an employee of the Wilhelminen Cancer Research Institute. He has received previous research support from Takeda and Amgen. He has also been a Speaker or had Advisory Board Honoraria from Celgene, Bristol‐Meyers, Takeda, Janssen, Amgen, and Sanofi. He received no direct benefit from any of these companies in relation to this specific manuscript. A.B., K.K., and A.R. are employees of Adelphi Real World. A.S. is an employee of Amgen, and received no direct benefit in relation to this manuscript. A.M. and K.B.C. were Amgen employees at the time of the study but are no longer Amgen employees, and received no direct benefit in relation to this manuscript. Amgen Ltd, who funded the analysis reported in this study, did not influence the original survey through either contribution to the design of questionnaires or data collection. Publication of study results was not contingent on the subscriber's approval or censorship of the manuscript.

## AUTHOR CONTRIBUTIONS

*Conceptualization and writing‐review & editing*, H.L.; *Conceptualization, data curation, investigation, methodology, project administration, resources, visualization and writing‐review and editing*, A.L.B; *Conceptualization, methodology, supervision, and writing‐review and editing*, A.M; *Conceptualization, data curation, investigation, methodology, project administration, resources, visualization and writing‐review and editing*, K.K.; *Data curation, formal analysis, software, validation and writing‐review and editing*, G.M.; *Validation, writing‐review and editing*, K.B.C.; *Conceptualization, investigation, methodology, project administration, resources, supervision, writing‐review and editing*, A.R.; *Conceptualization, funding acquisition, methodology, project administration, resources, supervision, writing‐review and editing*, A.S.

## ETHICS STATEMENT

Physicians consented to participate and provide patient medical information during screening. Patients also provided informed consent prior to completion of questionnaires, with all data aggregated and de‐identified before receipt. DSP data were collected according to procedures established at Adelphi Real World and are compliant with the European Pharmaceutical Market Research Association (EphMRA) Code of Conduct, the Health Information Technology for Economic and Clinical Health (HITECH) Act^16^ and the Health Insurance Portability and Accountability Act (HIPAA)^17^ as appropriate in each specific region or territory. International approval for the survey was also granted by the Freiburger Ethik Kommission International (FEKI (017/1791).

## Supporting information


**Appendix S1**: Supporting information.

## Data Availability

Data collection was undertaken by Adelphi Real World as part of an independent survey, entitled the Adelphi Real World Multiple Myeloma Disease Specific Programme. Amgen Ltd was a subscriber to this survey, who funded the analysis described in this study using data obtained from this survey. Amgen Ltd did not influence the original survey through either contribution to the design of questionnaires or data collection. All data that support the findings of this study are the intellectual property of Adelphi Real World. All requests for access should be addressed directly to Abigail Lucy Bailey (Adelphi Real World Lead Author) at abigail.bailey@adelphigroup.com
