# Supplementary material for: Patient‐reported pain severity and health‐related quality of life in patients with multiple myeloma in real world clinical practice
Source: Cancer Rep (Hoboken). 2021 Jun 10;5(1):e1429. doi: 10.1002/cnr2.1429 (PMC8789609; doi:10.1002/cnr2.1429)
Supplement: Supplementary file 1 — Appendix S1: Supporting information. [file CNR2-5-e1429-s001.docx]

| Alignment between single pain question and EQ-5D-5L | | | | | |
| --- | --- | --- | --- | --- | --- |
| **EQ-5D-5L**  **pain/discomfort severity** | **Single question: pain severity** | | | | |
|  | All patients | No | Mild | Moderate | Severe |
| n | 330 | 73 | 161 | 81 | 15 |
| No | 82 (25) | 69 (95) | 12 (7) | 1 (1) |  |
| Slight | 148 (45) | 3 (4) | 136 (84) | 9 (11) |  |
| Moderate | 81 (25) | 1 (1) | 13 (8) | 66 (81) | 1 (7) |
| Severe/Extreme | 19 (6) |  |  | 5 (6) | 14 (93) |
| All values are n (%) unless otherwise indicated.  Blank cells indicate that no patients were in that particular category. | | | | | |

| Alignment between single pain question and EORTC QLQ-MY20 | | | | | | |
| --- | --- | --- | --- | --- | --- | --- |
| **EORTC QLQ**  **MY20**^a^ | **Single question: pain severity** | | | | | |
|  | All patients | No pain | Mild pain | Moderate pain | Severe pain | ***p-*value**^b^ |
| n | 328 | 73 | 161 | 79 | 15 |  |
| Mean (SD) | 2.12 (0.79) | 1.38 (0.54) | 2.05 (0.68) | 2.76 (0.49) | 3.0 (0.65) | <0.0001 |
| Abbreviations: SD, standard deviation.  ^a^Bone aches or pain based off question 31 from EORTC QLQ-MY20; a mean score of 1 corresponds to “not at all” for no pain experienced in the past week, a mean score of 2 for “a little bit”, and a mean score of 3 for “quite a bit”  ^b^Based off Spearman's rank correlation coefficient from regression model | | | | | | |

## Table S1. Validated questionnaires for outcome data collection

|  | **Questionnaire** | **Description and scoring** |
| --- | --- | --- |
| Health-related quality of life | EORTC QLQ-C30  (Coordinator Q, 2001) | - Generic health-related quality of life questionnaire for cancer patients - Relevant scales for the current study: one global health status/quality of life scale, functional scales (physical, role, emotional, social functioning), symptom scales (fatigue, pain) - 4-point Likert-type scale (1 – not at all to 4 – very much), except for two 7-point global health status/status/quality of life items; scales and single-item measures range in score from 0 to 100 - A high score on global quality of life scales represent high quality of life - A high score on symptom scales corresponds to a high symptom burden - A high score on the functional scale corresponds to a high level of functioning |
|  | EORTC QLQ-M20  (add-on to the  EORTC-QLQ-C30)  (Coordinator Q, 2001) | - Myeloma-specific symptoms and functional impact questionnaire - Relevant scales for the current study: symptom subscale for disease symptoms, functional subscale on future perspectives - 4-point Likert-type scale (1 – not at all to 4 – very much); scales and single-item measures range in score from 0 to 100 - A high score on global quality of life scales represent high quality of life - A high score on symptom scales corresponds to a high symptom burden - A high score on the functional scale corresponds to a high level of functioning |
| Work productivity loss | WPAI  (Reilly Associates, 2002) | - Generic work productivity and activity impairment questionnaire - Outcomes are expressed as impairment percentages, that is a combination of absenteeism and presenteeism. - A high impairment percentage corresponds to a high level of impairment and a low level of work productivity |

| **Table S2. Association between self-reported pain severity and self-reported overall HRQoL in the past 7 days up to/at face-to-face clinical consultation (linear regression)** | | | | | | | | | | | | | | | | |
| --- | --- | --- | --- | --- | --- | --- | --- | --- | --- | --- | --- | --- | --- | --- | --- | --- |
| **Summary of bivariate regressions** | **Patient reported symptoms** | | | | | | | | | | | | | | | |
|  | **Overall** | | **No pain** | | **Mild pain** | | | | **Moderate pain** | | | | **Severe pain** | | | |
|  | n | *P*-value | n | mean | n | mean diff | 95% CI | *P*-value | n | mean diff | 95% CI | *P*-value | n | mean diff | 95% CI | *P*-value |
| **Overall health status** | | | | | | | | | | | | | | | | |
| EORTC QLQ-C30 Global health status | 330 | <0.0001 | 73 | 70.21 | 161 | -9.13 | -13.38,-4.88 | <0.0001 | 81 | -23.6 | -28.40,-18.80 | <0.0001 | 15 | -36.87 | -45.30,-28.44 | <0.0001 |
| **Overall work productivity loss** | | | | | | | | | | | | | | | | |
| WPAI: Percent work productivity loss due to problem | 35 | 0.0613 | 13 | 12.59 | 19 | 4.61 | -15.70,24.92 | 0.6472 | 3 | 43.32 | 7.18,79.46 | 0.0203 |  | | | |
| WPAI: Percent work time missed due to problem | 42 | 0.0405 | 16 | 23.13 | 23 | 8.61 | -4.95,22.17 | 0.2064 | 3 | 33.54 | 7.33,59.75 | 0.0135 |  |  |  |  |
| WPAI: Percent overall work impairment due to problem | 33 | 0.0342 | 12 | 23.5 | 19 | 18.57 | -1.88,39.02 | 0.0734 | 2 | 51.66 | 9.30,94.01 | 0.0185 |  |  |  |  |
| **Overall function impairment** | | | | | | | | | | | | | | | | |
| WPAI: Percent activity impairment due to problem | 319 | <0.0001 | 71 | 35.35 | 159 | 6.85 | 1.06,12.64 | 0.0192 | 75 | 19.58 | 12.94,26.22 | <0.0001 | 14 | 36.08 | 24.35,47.81 | <0.0001 |
| EORTC QLQ-C30: Role functioning (RF2) | 329 | <0.0001 | 73 | 79.45 | 160 | -17.47 | -23.52,-11.43 | <0.0001 | 81 | -29.86 | -36.75,-22.98 | <0.0001 | 15 | -40.56 | -52.66,-28.46 | <0.0001 |
| EORTC QLQ-C30: Physical functioning (PF2) | 330 | <0.0001 | 73 | 82.74 | 161 | -11.15 | -16.11,-6.18 | <0.0001 | 81 | -28.4 | -33.96,-22.84 | <0.0001 | 15 | -47.63 | -57.40,-37.86 | <0.0001 |
| EORTC QLQ-C30: Fatigue (FA) | 330 | <0.0001 | 73 | 25.95 | 161 | 10.18 | 4.56,15.79 | 0.0004 | 81 | 28.64 | 22.28,35.01 | <0.0001 | 15 | 42.94 | 31.75,54.12 | <0.0001 |
| EORTC QLQ-C30: Social functioning (SF) | 329 | <0.0001 | 73 | 81.05 | 160 | -4.9 | -11.27,1.46 | 0.1304 | 81 | -15.82 | -22.99,-8.66 | <0.0001 | 15 | -36.61 | -49.19,-24.02 | <0.0001 |
| EORTC QLQ-C30: Emotional functioning (EF) | 327 | <0.0001 | 73 | 78.08 | 158 | -9.34 | -14.61,-4.08 | 0.0005 | 81 | -22.32 | -28.32,-16.33 | <0.0001 | 15 | -29.75 | -40.28,-19.22 | <0.0001 |
| Each coefficient will indicate how the related group differs from the base case (no pain) | | | | | | | | | | | | | | | | |
